# Effects of Intermittent Compared With Continuous Energy Restriction on Blood Pressure Control in Overweight and Obese Patients With Hypertension

**DOI:** 10.3389/fcvm.2021.750714

**Published:** 2021-10-18

**Authors:** Chao-Jie He, Ye-Ping Fei, Chun-Yan Zhu, Ming Yao, Gang Qian, Hui-Lin Hu, Chang-Lin Zhai

**Affiliations:** ^1^Department of Cardiology, The First Hospital of Jiaxing, The Affiliated Hospital of Jiaxing University, Jiaxing, China; ^2^Department of Anesthesiology, The First Hospital of Jiaxing, The Affiliated Hospital of Jiaxing University, Jiaxing, China

**Keywords:** hypertension, obesity, weight loss, 5:2 diet, intermittent energy restriction

## Abstract

**Background and Aims:** Weight-loss diets reduce body weight and improve blood pressure control in hypertensive patients. Intermittent energy restriction (IER) is an alternative to continuous energy restriction (CER) for weight reduction. We aimed to compare the effects of IER with those of CER on blood pressure control and weight loss in overweight and obese patients with hypertension during a 6-month period.

**Methods:** Two hundred and five overweight or obese participants (BMI 28.7 kg/m^2^) with hypertension were randomized to IER (5:2 diet, a very-low-calorie diet for 2 days per week, 500 kcal/day for women and 600 kcal/day for men, along with 5 days of a habitual diet) compared to a moderate CER diet (1,000 kcal/day for women and 1,200 kcal/day for men) for 6 months. The primary outcomes of this study were changes in blood pressure and weight, and the secondary outcomes were changes in body composition, glycosylated hemoglobin A1c (HbA1c), and blood lipids.

**Results:** Of the 205 randomized participants (118 women and 87 men; mean [SD] age, 50.2 [8.9] years; mean [SD] body mass index, 28.7 [2.6]; mean [SD] systolic blood pressure, 143 [10] mmHg; and mean [SD] diastolic blood pressure, 91 [9] mmHg), 173 completed the study. The intention-to-treat analysis demonstrated that IER and CER are equally effective for weight loss and blood pressure control: the mean (SEM) weight change with IER was −7.0 [0.6] kg vs. −6.8 [0.6] kg with CER, the mean (SEM) systolic blood pressure with IER was −7 [0.7] mmHg vs. −7 [0.6] mmHg with CER, and the mean (SEM) diastolic blood pressure with IER was −6 [0.5] mmHg vs. −5 [0.5] mmHg with CER, (diet by time *P* = 0.62, 0.39, and 0.41, respectively). There were favorable improvements in body composition, HbA1c, and blood lipid levels, with no differences between groups. Effects did not differ according to completer analysis. No severe hypoglycemia occurred in either group during the trial.

**Conclusions:** Intermittent energy restriction is an effective alternative diet strategy for weight loss and blood pressure control and is comparable to CER in overweight and obese patients with hypertension.

**Clinical Trial Registration:** http://www.chictr.org.cn, identifier: ChiCTR2000040468.

## Introduction

Overweight and obesity have become major global public health concerns over the past few decades. The prevalence of obesity is increasing in Western societies, as well as developing countries, and is likely to increase the burden of chronic health conditions, including hypertension, coronary heart disease and diabetes, and consequently cardiovascular morbidity and mortality (1, 2). The latest World Health Organization global estimates showed that about 1.9 billion adults were overweight, with 650 million of these obese in 2005, and if the recent secular trends continue unabated, it is predicted that about 2.16 billion and 1.12 billion individuals will be, respectively overweight or obese by 2,030 (3, 4). Obesity was estimated to affect 49.5% of US adults with high blood pressure (BP) in 2012; similarly, half of all hypertensive patients were overweight or obese in China in the same year (5, 6).

Lifestyle modifications, including weight loss and increased physical activity, are recommended in major guidelines as a first-step intervention in the treatment of hypertensive patients (7). A meta-analysis of randomized controlled trials showed reductions in systolic blood pressure (SBP) and diastolic blood pressure (DBP) of ≈1 mmHg for each kilogram of weight loss (8). Effective energy restriction strategies are required, and continuous energy restriction (CER) is widely employed for weight management (9). Part of the difficulty with weight loss and maintenance by low-calorie diets is that the body responds to CER through a series of compensatory changes in biological and behavioral determinants of body composition. Resting energy expenditure has been shown to decrease to a greater degree than that expected from changes in body composition during continuous calorie restriction, a physiological phenomenon termed “adaptive thermogenesis” that has been observed in rodents and humans during fasting and severe energy restriction (10–12). This results in lower efficiency of weight reduction. More recently, intermittent energy restriction (IER) has drawn attention in the popular media as an alternative to CER due to its feasibility and even potential for higher rates of compliance (13). The two most popular forms of IER are alternate-day fasting and the 5:2 eating pattern, the latter characterized by a very low-calorie diet (500 kcal/day for women and 600 kcal/day for men) for 2 days per week, consisting of cycles of restriction dotted with periods of deliberate energy balance, which may attenuate compensatory responses associated with CER, and thus provide an alternative strategy to achieve and maintain weight loss in place of traditional approaches (14, 15).

The Dietary Approaches to Stop Hypertension (DASH) diet is recommended for adults with elevated BP to achieve a desirable weight (5). A recent meta-analysis of 13 randomized controlled clinical trials by Soltani et al., revealed that the DASH diet had produced an approximate overall weight reduction of 1.42 kg and SBP reduction of 11 mmHg in 8–24 weeks, particularly for overweight and obese subjects (16). Besides, Semlitsch et al. have shown that continuous weight-loss diets reduced body weight and BP in individuals with primary hypertension (17). Although the DASH diet and sustainable energy restriction have been demonstrated to reduce BP and weight, no trials have examined the effects of IER in overweight and obese individuals with high BP (5, 8). The objective of this randomized trial was to determine the effects of the 5:2 diet compared with CER on BP and weight loss at 6 months in overweight and obese patients with hypertension. We hypothesized that equal improvements to BP and weight loss would occur at 6 months. Secondary outcomes included examining if there were differences in body composition, glycosylated hemoglobin A1c (HbA1c), and blood lipid levels.

## Methods

### Study Population and Design

This was a parallel, randomized clinical trial conducted at the Affiliated Hospital of Jiaxing University from June 1, 2020, to April 30, 2021. The study was approved by the Ethics Committee of Jiaxing University based on the Helsinki declaration (protocol number LS2020-311) and registered retrospectively 3 months after commencement of the study with the Chinese Clinical Trial Registry (ChiCTR2000040468). All participants gave informed consent prior to recruitment and were provided compensation in the form of a $38 voucher at 3 and 6 months for their time for participating in the study. The Academic Committee was responsible for security and privacy supervision, including the design of the protocol. Participants were recruited using advertisements and flyers posted in the hospital and local communities.

### Participants and Randomization

The main inclusion criteria were age between 18 and 70 years, hypertension, and body mass index (BMI, calculated as weight in kilograms divided by height in meters squared) ranging from 24 to 40 kg/m^2^. The exclusion criteria were SBP ≥180 mmHg or DBP ≥120 mmHg, type 1 or 2 diabetes with a history of severe hypoglycemic episodes, pregnancy or breastfeeding, usage of glucagon-like peptide 1 receptor agonists, weight loss >5 kg within the past 3 months or previous weight loss surgery, and inability to adhere to the dietary protocol. Of the 294 participants screened for eligibility, 205 were randomized 1:1 to the treatment groups, stratified by BMI and sex (as overweight or obese). Moreover, all participants were required to have a stable medication regimen and weight in the 3 months before enrollment and not to use weight-loss drugs or vitamin supplements for the duration of the study. Researchers and participants were not blinded to the study group assignment.

### Dietary Intervention

Participants randomly assigned to the IER group followed a 5:2 eating pattern (a very-low-energy diet of 500–600 kcal for 2 days of the week along with their usual diet for the other 5 days. The 2 days of calorie restriction could be consecutive or non-consecutive, with a minimum of 0.8 g supplemental protein per kilogram of body weight per day, in accordance with the Dietary Guidelines for Chinese Residents (2016) (18). An example meal plan can be found in Supplementary Table 1 in the supplementary material. The CER group was advised to consume 1,000 kcal/day for women and 1,200 kcal/day for men on a 7-day energy restriction. That is, they were prescribed a daily 25% restriction based on the general principles of a Mediterranean-type diet (30% fat, 45–50% carbohydrate, and 20–25% protein) (19). Both groups received dietary education from a qualified dietitian and were recommended to maintain their current daily activity levels throughout the trial. Written dietary information brochures with portion advice and sample meal plans were provided to improve compliance in each group. All participants received a digital cooking scale (Blone digital kitchen scale; PR-SYDZC1, Germany, accuracy: 0.1 g) to weigh foods to ensure accuracy of intake and were required to keep a food diary while following the recommended recipe on 2 days/week during calorie restriction to help with adherence. No food was provided. All participants were followed up by regular outpatient visits to both cardiologists and dietitians once a month. Diet checklists, activity schedules, and weight were reviewed to assess compliance with dietary advice at each visit.

### Medication Management

Participants were encouraged to measure and record their BP twice daily, and if two consecutive BP readings were <110/70 mmHg and/or with hypotensive episodes with symptoms (dizziness, nausea, headache, and fatigue), they were asked to contact the investigators directly. Antihypertensive medication changes were then made at clinic visits, by telephone, or by WeChat, in consultation with cardiologists.

The medication management protocol was designed to avoid hypoglycemia after a review of the relevant literature and consultation with an endocrinologist for patients with diabetes. The doses of antidiabetic medications, including insulin and sulfonylurea, could be reduced to avoid severe hypoglycemia. Medication could be reduced in the CER diet group based on the basal dose at the endocrinologist's discretion. As for the IER group, insulin and sulfonylureas were discontinued on calorie restriction days only, and long-acting insulin was discontinued the night before the IER day (20). Insulin was not to be resumed until a full day's caloric intake was achieved.

### Outcome Measures

All outcome measures were recorded at baseline and at each monthly visit. The primary outcomes of this study were changes in BP and weight, and the secondary outcomes were changes in body composition, HbA1c, and blood lipids after 6 months.

BP was measured three times on the participants' right upper arm after 5 min of seated rest using an automatic digital sphygmomanometer (Omron HEM-7124, Omron Corporation, Kyoto, Japan). Bodyweight was measured to the nearest 0.1 kg at all six visits in the fasting state using an electronic scale (Omron, HN-289-W), while height was recorded to the nearest 0.1 cm at baseline only. All individuals were asked to be barefoot or in light footwear and to wear light clothing during these measurements. Body composition was assessed by dual-energy X-ray absorptiometry scanning (DEXA, Discovery Wi/HOLOGIC Inc., Bedford, USA) by a licensed radiation technician. All female participants of reproductive age were asked if they might be pregnant, as a urinary pregnancy test would precede body composition assessment; however, no pregnancy tests were needed during the study period. Blood samples were collected from a brachial vein after a 12-h overnight fast for measurements of lipid levels, HbA1c, and fasting blood glucose. Serum was separated by centrifugation (+4°C, 4,000 rpm, 10 min) and then stored frozen at −80°C for further analysis.

The incidence rates of hypoglycemia were based on blood glucose (defined as BG <70 mg/dL) and/or symptomatic hypoglycemia (symptoms of sweating, paleness, dizziness, and confusion). Two cardiologists who were blind to the patients' diet condition measured and recorded all pertinent clinical parameters and adjudicated serious adverse events.

### Statistical Analysis

The sample size calculation was based on a mean 5.0 kg (SD 4.9 kg) reduction in body weight after 12 months in both groups from a previous study (20). We required a minimum sample of 164 individuals to have 80% power to demonstrate a 2.5 kg difference in weight loss between groups with an α of 0.05. For BP, a very similar number was needed to detect a 5-mm Hg difference in SBP between treatment arms. A weight loss of 2.5 kg and a decrease in SBP by 5 mmHg correlates with clinically significant reductions in coronary artery disease and stroke (21). To take into account loss to follow-up, we enrolled a final number of 205 participants.

We performed our analyses both on the basis of completers and on an intention-to-treat principle. Continuous data are presented as mean ± SD or mean ± standard error of the mean (SEM) with a two-sided 95% CI and compared using independent-samples *t*-tests or the Mann–Whitney *U* test. Categorical variables were summarized as frequency and percentage, and Pearson's Chi-square test or Fisher's exact test was applied as appropriate. We employed repeated-measures ANOVA via a linear mixed model to test the effects of diet, time, and their interaction. In subgroup analyses, we evaluated differential effects of the intervention on the primary outcomes with respect to patients' level of education, domicile and sex based on the statistical significance of the interaction term for the subgroup of interest in the multivariate model. *P* < 0.05 was considered statistically significant. All statistical analyses were conducted using IBM SPSS Statistics 23.0 software (IBM SPSS Statistics, IBM, Chicago, USA).

## Results

### Study Participants

**Figure 1:**
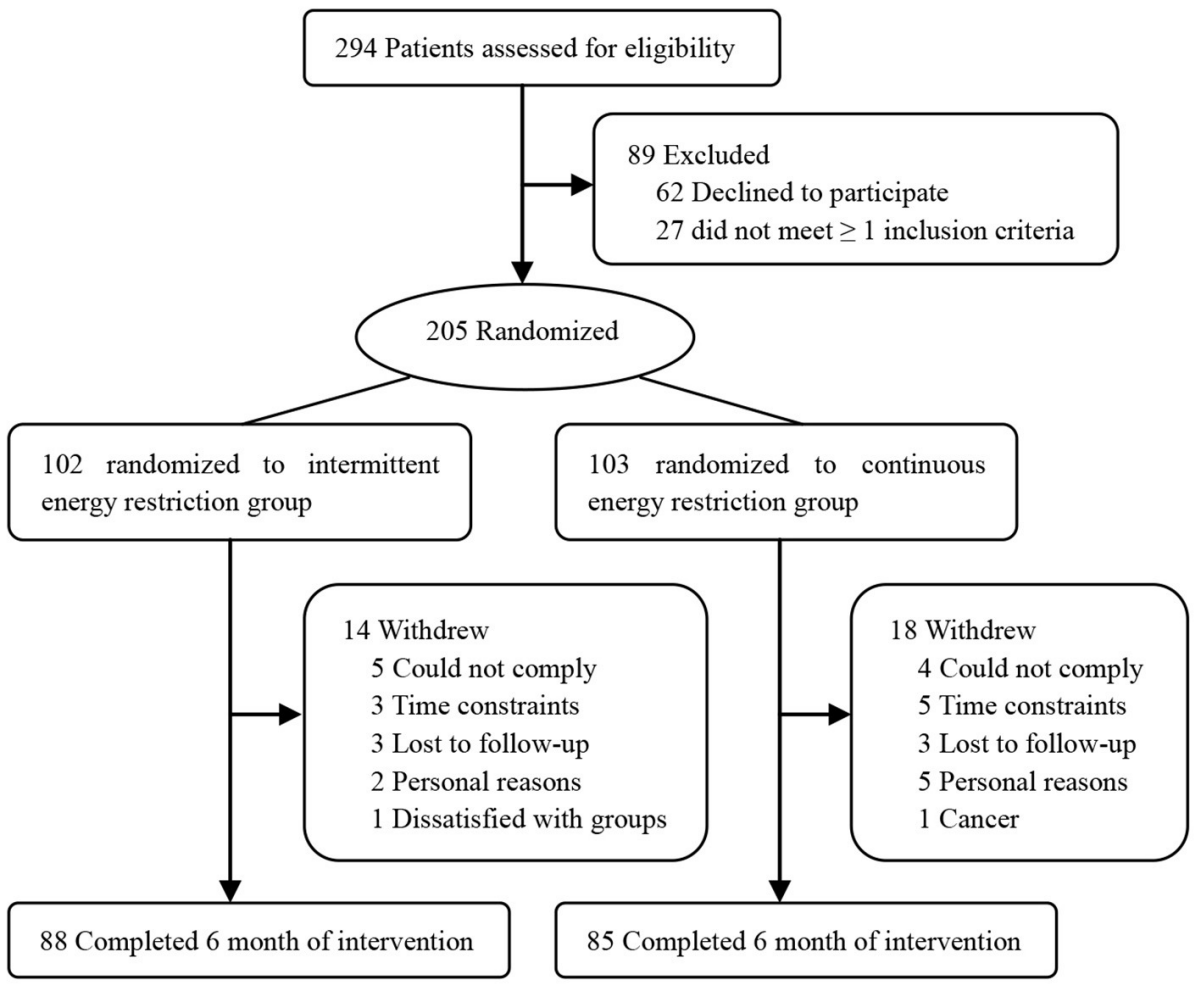
Participant flow diagram.

A total of 294 participants were screened, 205 Chinese adults with high BP [118 women and 87 men; mean (SD) age, 50.5 (8.8) years; mean (SD) BMI, 28.7 (2.6); mean (SD) SBP, 143 (10) mmHg; and mean (SD) DBP, 91 (9) mmHg] were randomly assigned to diet groups. By means of a randomized digital table, 102 subjects were individually randomized to the IER group, and 103 subjects were randomized to the CER group. One hundred and seventy-three participants (84.4%) completed the study (Figure 1), and both groups had similar dropout rates at 6 months [14 participants (13.7%) in the IER group and 18 participants (17.5%) in the CER group; *P* = 0.83]. Both groups were well matched for baseline characteristics except for triglyceride levels (Table 1). Participants were predominately middle-aged, overweight, had mildly to moderately elevated SBP and DBP, and lacked physical exercise as classified by BMI.

**Table 1:** Demographic and clinical characteristics of the study population.

	**Mean (SD) value**				
**Characteristic**	**IER (*n* = 102)**	**CER (*n* = 103)**	**All participants**	**Participants who**	**Participants**	***p*-value**
				**completed**	**who did not**	
			**(*N* = 205)**	**study (*n* = 173)**	**complete study (*n* = 32)**	
**Demographics**						
Age, y	50.2 (8.9)	50.7 (8.7)	50.5 (8.8)	50.1 (8.8)	52.8 (8.9)	0.53
Female, *n* (%)	58 (56.9)	60 (58.3)	118 (57.6)	97 (56.1)	21 (65.6)	0.55
Overweight, *n* (%)	65 (63.7)	64 (62.1)	129 (62.9)	110 (63.6)	19 (59.4)	0.78
Obese, *n* (%)	37 (36.3)	39 (37.9)	76 (37.1)	63 (36.4)	13 (40.6)	0.63
Duration of HBP, y	6.8 (5.9)	6.6 (5.7)	6.7 (5.8)	6.7 (5.8)	6.7 (5.7)	0.59
**Comorbid condition**, ***n*** **(%)**						
Diabetes	32 (31.4)	29 (28.2)	61 (29.8)	50 (28.9)	12 (37.5)	0.39
Hyperlipidemia	71 (69.6)	73 (70.9)	144 (70.2)	122 (70.5)	22 (68.8)	0.69
OSAHS	38 (37.3)	42 (40.8)	80 (39.0)	68 (39.3)	12 (37.5)	0.40
**Blood pressure at baseline (mmHg)**						
SBP	143 (10)	144 (10)	143 (10)	143 (10)	144 (10)	0.72
DBP	91 (9)	91 (8)	91 (9)	91 (9)	91 (9)	0.66
**Antihypertensive drugs**, ***n*** **(%)**						
RAAS inhibitors	69 (67.6)	70 (68.0)	139 (67.8)	120 (69.4)	19 (59.4)	0.88
CCB	64 (62.7)	67 (62.1)	131 (63.9)	114 (65.9)	17 (53.1)	0.76
Diuretic	50 (49.0)	49 (47.6)	99 (48.3)	81 (46.8)	17 (53.1)	0.89
β-Blockers	54 (52.9)	55 (53.4)	109 (53.2)	89 (51.4)	20 (62.5)	0.90
**Body weight and composition**						
Weight, kg	86.2 (14.2)	86.4 (14.1)	86.3 (14.1)	86.2 (14.3)	86.8 (14.0)	0.68
BMI	28.7 (2.6)	28.7 (2.5)	28.7 (2.7)	28.6 (2.6)	28.8 (2.7)	0.97
Waist circumference, cm	97.6 (8.1)	98.3 (8.2)	98.0 (8.1)	97.9 (8.0)	98.4 (8.3)	0.63
Total body fat, %	40.1 (6.3)	40.3 (6.2)	40.2 (6.3)	40.1 (6.1)	40.6 (6.3)	0.77
Total fat mass, kg	33.7 (7.9)	33.9 (8.1)	33.8 (8.1)	33.7 (8.0)	34.0 (8.1)	0.75
Android fat, %	49.4 (5.3)	49.6 (5.5)	49.5 (5.5)	49.5 (5.4)	49.5 (5.4)	0.80
Android fat mass, kg	4.3 (1.0)	4.3 (1.2)	4.3 (1.1)	4.2 (1.2)	4.5 (1.1)	0.85
**Laboratory parameters**						
Triglycerides, mmol/L	3.0 (1.5)	3.5 (1.6)	3.2 (1.5)	3.2 (1.6)	3.4 (1.5)	0.03
Total cholesterol, mmol/L	6.2 (1.2)	6.4 (1.3)	6.3 (1.3)	6.3 (1.4)	6.3 (1.3)	0.68
LDL cholesterol, mmol/L	4.0 (1.0)	3.9 (1.0)	4.0 (0.9)	3.9 (1.1)	4.0 (1.0)	0.63
HDL cholesterol, mmol/L	0.9 (0.3)	0.9 (0.2)	0.9 (0.3)	0.9 (0.3)	1.0 (0.3)	0.89
HbA1c, %	6.3 (1.2)	6.2 (1.3)	6.2 (1.2)	6.2 (1.2)	6.3 (1.3)	0.63
FPG, mmol/L	7.1 (1.6)	7.0 (1.4)	7.0 (1.6)	7.0 (1.5)	7.2 (1.6)	0.62
**Physical activity**, ***n*** **(%)**						
≤ 2 h/wk	72 (70.6)	70 (68.0)	142 (69.3)	120 (69.4)	22 (68.8)	0.67
>2 h/wk	30 (29.4)	33 (32.0)	63 (30.7)	43 (30.6)	10 (31.2)	0.67

*SD, indicates standard deviation; IER, intermittent energy restriction; CER, continuous energy restriction; HBP, high blood pressure; OSAHS, obstructive sleep apnea hypopnea syndrome; SBP, systolic blood pressure; DBP, diastolic blood pressure; RAAS, rennin-angiotensin-aldosterone system; CCB, calcium channel blockers; MES, medication effect score; BMI, body mass index; LDL, low-density lipoprotein; HDL, high-density lipoprotein; HbA1c, hemoglobin A1c; FPG, fasting plasma glucose*.

### Weight Loss and Blood Pressure

**Figure 2:**
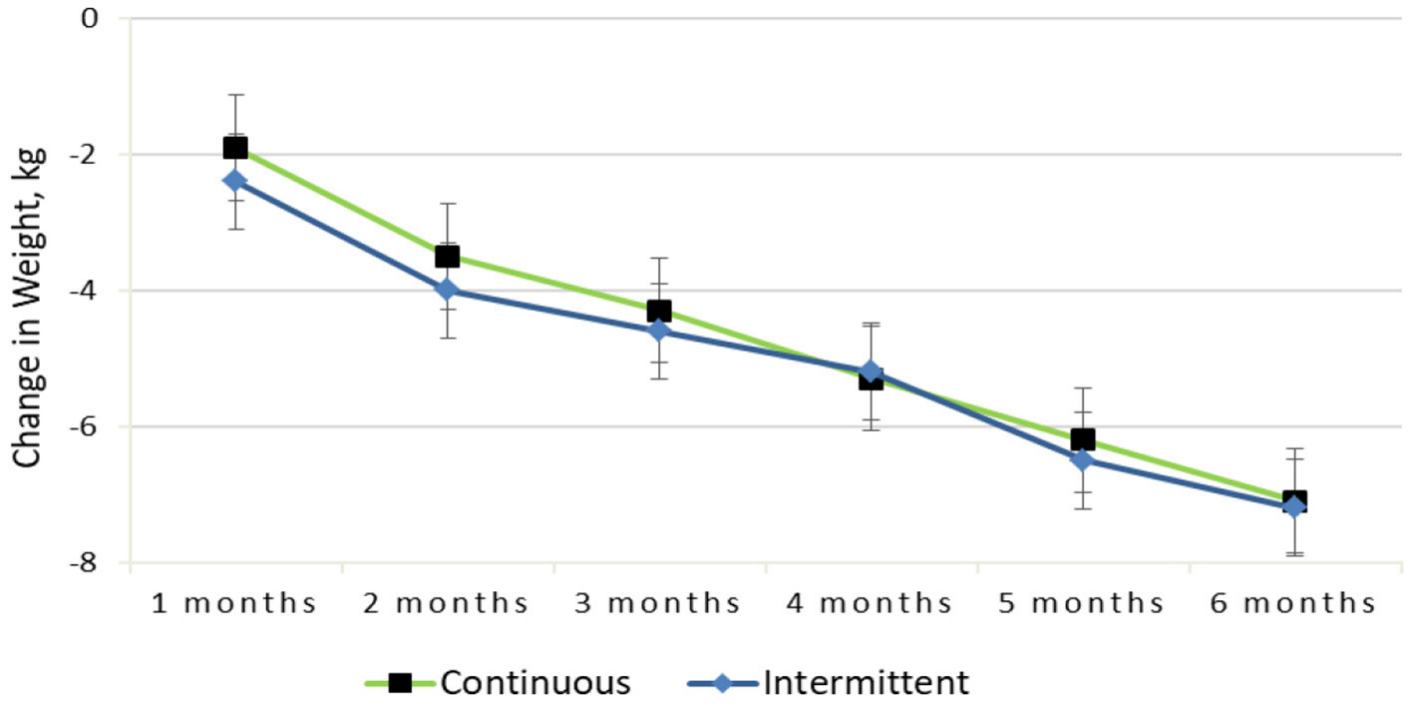
Change in weight from baseline to 6-months for the continuous vs. intermittent groups (completer analysis).

From baseline to 6 months, both groups experienced significant reductions in weight [mean (SEM)], and there was no difference between treatment groups [−7.2 (0.6) kg in the IER group vs. −7.1 (0.6) kg in the CER group; diet by time *P* = 0.72]. Figure 2 shows that both dietary interventions followed a similar weight loss pattern throughout the trial. Fifty-one participants lost ≥10% of their baseline body weight, and the remaining 79 subjects lost between 5 and 10% at 6 months. Notably, obese participants had the greatest weight change [mean (SEM), −9.1 (0.8) kg; *P* < 0.01], and overweight participants had a smaller change [−5.0 (0.5) kg; *P* < 0.01] at 6 months.

**Figure 3:**
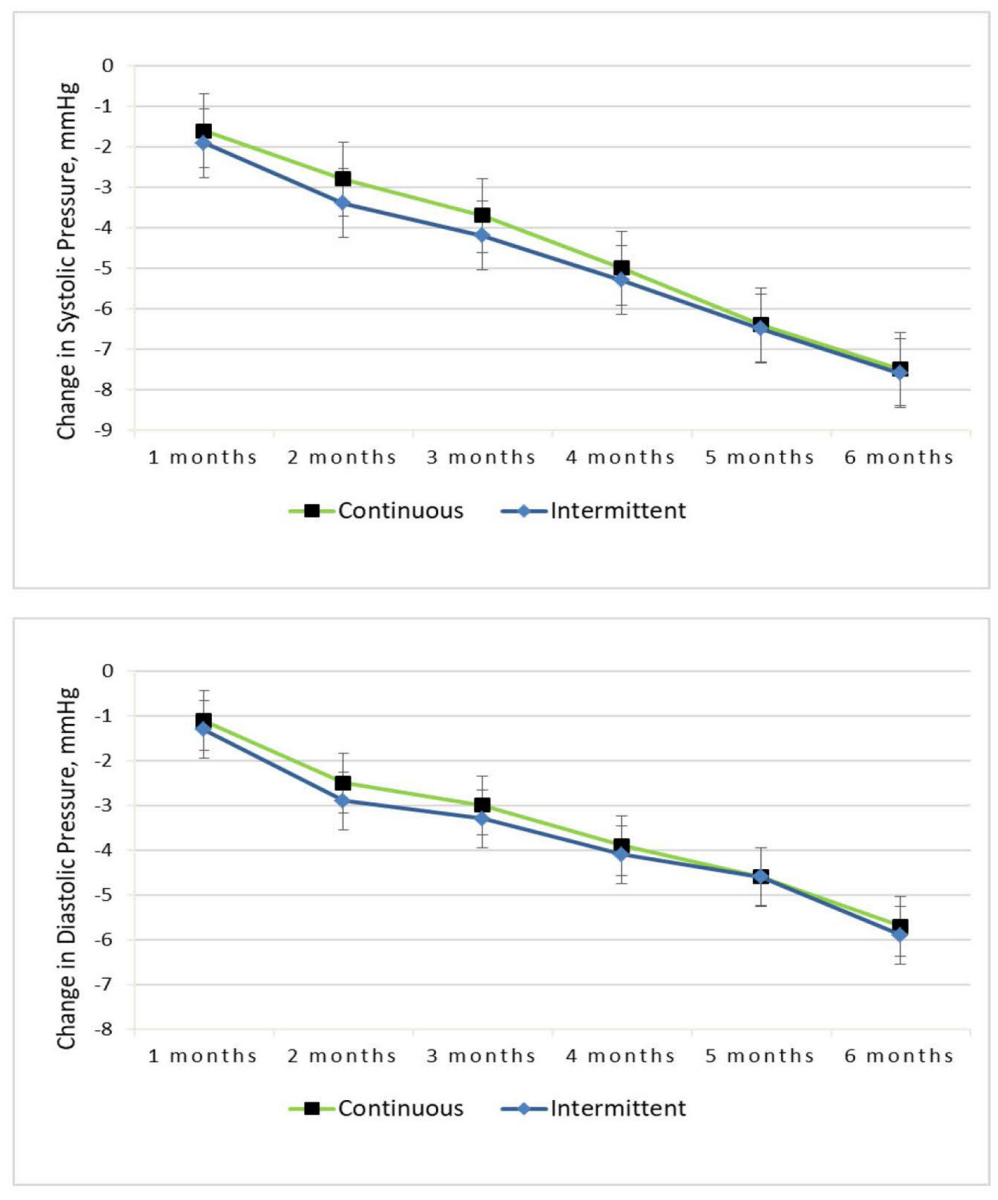
Change in systolic and diastolic pressure from baseline to 6-months for the continuous vs. intermittent groups (completer analysis).

BP reduction in both arms is highlighted in Figure 3. The change in SBP and DBP achieved was statistically significant over time, and there was no difference between the dietary interventions [−8 (0.7) mmHg in the IER group vs. −8 (0.6) mmHg in the CER group, diet by time *P* = 0.68; −6 (0.6) mmHg in the IER group vs. −6 (0.5) mmHg in the CER group, diet by time *P* = 0.53]. A similar pattern was seen for SBP (SEM), and the obese subgroup had a decrease of −8 [0.7] mmHg, compared with a reduction of −7 [0.6] mmHg in the overweight group.

In the subgroup by sex, both groups experienced significant reductions in weight, SBP and DSP [mean (SEM)], and there was no difference between groups [−7.1 (0.5) kg in the female group vs. −7.3 (0.6) kg in the male group; diet by time *P* = 0.40, −8 (0.6) mmHg in the female group vs. −7 (0.6) mmHg in the male group, diet by time *P* = 0.52; −6 (0.5) mmHg in the female group vs. −6 (0.5) mmHg in the male group, diet by time *P* = 0.33]. Subgroup analyses of the association of the intervention with weight, SBP and DBP by education (“middle school degree or below”, “high school degree”, “college degree or above”) and domicile (country or city) showed no significant between-group differences.

### Body Composition, HbA1c, and Blood Lipids

Changes in body composition, HbA1c, and blood lipids are presented in Table 2. All measures of body composition decreased significantly at 6 months; both groups experienced comparable reductions in total fat mass [mean (SEM)], and android fat mass [−5.5 (0.6) kg in the IER group vs. −4.8 (0.5) kg in the CER group, diet by time *P* = 0.08; −1.1 (0.2) kg in the IER group vs. −0.8 (0.2) kg in the CER group, diet by time *P* = 0.16]. Of note, participants in the CER group lost significantly more total fat-free mass than did participants in the IER group [mean (SEM), −2.3 (0.2) kg vs. −1.7 (0.2) kg; *P* = 0.03], and there was a trend toward a greater change in total fat mass in the intermittent calorie restriction group (*P* = 0.08). The secondary outcome of mean (SEM) HbA1c [−0.2% (0.1%)] and blood lipid levels [triglyceride level, −1.0 (0.3) mmol/L; total cholesterol level, −0.9 (0.2) mmol/L; low-density lipoprotein cholesterol level, −0.9 (0.2) mmol/L; high-density lipoprotein cholesterol level, 0.7 (0.3) mmol/L] improved with weight loss (*P* < 0.05), and there were no differences between groups (diet by time *P* > 0.05).

**Table 2:** Primary and secondary outcomes from baseline to 6 months for IER vs. CER groups (completers analysis).

**Variable**	**Mean (SEM) [95% CI]**	***p*-value (time)**	**Mean (SEM) [95% CI]**		***p*-value (diet × time)**
	**All participants (*n* = 173)**		**IER group (*n* = 88)**	**CER group (*n* = 85)**	
**Primary outcomes**					
Systolic BP, mmHg	−8 (0.7) [−11-−4]	<0.001	−8 (0.7) [−10-−4]	−8 (0.6) [−10-−4]	0.68
Diastolic BP, mmHg	−6 (0.6) [−10-−3]	<0.001	−6 (0.6) [−9-−4]	−6 (0.5) [−8-−4]	0.53
Weight, kg	−7.2 (0.6) [−11.2-−3.8]	<0.001	−7.2 (0.6) [−11.1-−4.1]	−7.1 (0.6) [−10.8-−3.7]	0.72
**Secondary outcomes**					
Total body fat, %	−1.7 (0.3) [−2.4-−1.1]	<0.001	−1.9 (0.3) [−2.5-−1.0]	−1.6 (0.3) [−2.3-−0.6]	0.32
Total fat mass, kg	−5.2 (0.5) [−6.1-−3.9]	<0.001	−5.5 (0.6) [−5.6-−3.4]	−4.8 (0.5) [−4.4-−2.4]	0.08
Total fat-free mass, kg	−2.0 (0.3) [−2.7-−1.3]	<0.001	−1.7 (0.2) [−2.6-−1.0]	−2.3 (0.2) [−2.7-−0.9]	0.03
Android fat, %	−3.6 (1.0) [−6.1-−1.7]	<0.001	−3.9 (1.1) [−6.8-−2.2]	−3.3 (1.0) [−5.9-−1.5]	0.29
Android fat mass, kg	−0.9 (0.2) [−1.4-−0.5]	<0.001	−1.1 (0.2) [−1.8-−0.7]	−0.8 (0.2) [−1.3-−0.5]	0.16
Android free-fat mass, kg	−0.3 (0.1) [−0.6-−0.1]	<0.001	−0.3 (0.1) [−1.6-−0.1]	−0.3 (0.1) [−0.6-−0.07]	0.17
Triglycerides, mmol/L	−1.0 (0.3) [−1.9-−0.3]	<0.001	−0.8(0.3) [−1.4-−0.2]	−1.2 (0.3) [−2.0-−0.6]	0.36
Total cholesterol, mmol/L	−0.9 (0.2) [−1.8-−0.4]	<0.001	−0.8 (0.2) [−1.7-−0.4]	−1.0 (0.3) [−1.4-−0.5]	0.45
LDL cholesterol, mmol/L	−0.7 (0.3) [−1.1-−0.3]	<0.001	−0.9 (0.3) [−1.4-−0.3]	−0.6 (0.3) [−1.1-−0.3]	0.55
HDL cholesterol, mmol/L	0.1 (0.1) [0.02-0.23]	<0.05	0.1 (0.1) [0.01-0.21]	0.2 (0.1) [0.08-0.29]	0.63
HbA1c, %	−0.2 (0.1) [−0.3-−0.1]	<0.01	−0.2 (0.1) [−0.4-−0.1]	−0.2 (0.1) [−0.3-−0.1]	0.87

*SEM, indicates standard error of mean; IER, intermittent energy restriction; CER, continuous energy restriction; SD, standard deviation; BP, blood pressure; LDL, low-density lipoprotein; HDL, high-density lipoprotein; HbA1c, hemoglobin A1c*.

### Intention-to-Treat Analysis

An intention-to-treat analysis (ITT) was applied for the primary outcome variable of weight and BP at 6 months based on the last observation carried forward. ITT analysis (*n* = 205) indicated that there was a significant effect of the interventions on weight loss over time (*P* < 0.01), with no significant effect of treatment (diet by time *P* = 0.62). Similarly, both SBP and DBP decreased significantly at 6 months (*P* < 0.01), and there was no difference between groups (diet by time *P* = 0.39 and 0.41, respectively) (Table 3).

**Table 3:** Primary and secondary outcomes from baseline to 6 months for intermittent vs. continuous groups (intention-to-treat analysis).

**Variable**	**Mean (SEM) [95% CI]**	***p*-value (time)**	**Mean (SEM) [95% CI]**		***p*-value (diet × time)**
	**All participants (*n* = 205)**		**IER group (*n* = 102)**	**CER group (*n* = 103)**	
**Primary outcomes**					
Systolic BP, mmHg	−7 (0.7) [−11-−3]	<0.001	−7 (0.7) [−9-−4]	−7 (0.6) [−9-−4]	0.39
Diastolic BP, mmHg	−6 (0.5) [−10-−3]	<0.001	−6 (0.5) [−8-−4]	−5 (0.5) [−8-−4]	0.41
Weight, kg	−6.9 (0.6) [−11.0-−3.5]	<0.001	−7.0 (0.6) [−11.1-−4.1]	−6.8 (0.6) [−10.4-−3.5]	0.62
**Secondary outcomes**					
Total body fat, %	−1.6 (0.3) [−2.3-−1.1]	<0.001	−1.7 (0.3) [−2.4-−0.9]	−1.6 (0.3) [−2.3-−0.6]	0.33
Total fat mass, kg	−5.0 (0.5) [−6.0-−3.7]	<0.001	−5.4 (0.6) [−5.6-−3.4]	−4.7 (0.5) [−4.4-−2.4]	0.07
Total fat-free mass, kg	−1.9 (0.3) [−2.6-−1.3]	<0.001	−1.6 (0.2) [−2.6-−1.3]	−2.1 (0.2) [−2.6-−0.9]	0.04
Android fat, %	−3.5 (0.9) [−6.0-−1.7]	<0.001	−3.7 (1.1) [−6.6-−2.2]	−3.3 (1.0) [−5.7-−1.4]	0.44
Android fat mass, kg	−0.8 (0.2) [−1.3-−0.4]	<0.001	−1.0 (0.2) [−1.8-−0.6]	−0.7 (0.1) [−1.2-−0.5]	0.21
Android fat-free mass, kg	−0.3 (0.1) [−0.5-−0.1]	<0.001	−0.3 (0.1) [−1.6-−0.1]	−0.3 (0.1) [−0.6-−0.08]	0.13
Triglycerides, mmol/L	−1 (0.4) [−2.1-−0.3]	<0.001	−0.9(0.4) [−1.6-−0.2]	−1.1 (0.4) [−2.0-−0.5]	0.72
Total cholesterol, mmol/L	−0.9 (0.4) [−1.9-−0.4]	<0.001	−0.8 (0.4) [−1.7-−0.4]	−1.0 (0.3) [−1.4-−0.5]	0.65
LDL cholesterol, mmol/L	−0.7 (0.3) [−1.1-−0.3]	<0.001	−0.9 (0.3) [−1.4-−0.3]	−0.6 (0.3) [−1.1-−0.3]	0.59
HDL cholesterol, mmol/L	0.1 (0.1) [0.02-0.22]	<0.05	0.1 (0.1) [0.01-0.21]	0.2 (0.1) [0.07-0.28]	0.38
HbA1c, %	−0.2 (0.1) [−0.3-−0.1]	<0.01	−0.2 (0.1) [−0.4-−0.1]	−0.2 (0.1) [−0.3-−0.1]	0.81

*SEM, indicates standard error of mean; IER, intermittent energy restriction; CER, continuous energy restriction; SD, standard deviation; BP, blood pressure; LDL, low-density lipoprotein; HDL, high-density lipoprotein; HbA1c, hemoglobin A1c*.

### Adverse Events

No participants in either group experienced a serious adverse event, and only minor adverse events were reported. In the CER group, four participants reported fatigue, seven dizziness, six mild headache, and three nausea during the first month, while six participants reported fatigue, 10 dizziness, five mild headache, four nausea, and two sleep disturbance in the same period in the IER group. Thirteen participants (11 with diabetes) experienced a mild hypoglycemic event (five events in the CER group and eight events in the IER group; *P* = 0.09) throughout the trial. The insulin and/or sulfonylurea doses were adjusted for all these 11 diabetic patients in a timely manner. There was no death occurred during the study period and only one minor stroke in IER group.

## Discussion

The most commonly employed strategy for weight loss is daily calorie restriction, which involves reducing usual caloric intake on a daily basis by 15–60% (9). IER offers a reduced burden of calorie restriction and shows promise for achieving weight reduction goals; therefore, it may be a viable alternative to CER. To our knowledge, this is the first randomized controlled trial to explore the two forms of energy restriction, indicating that IER is as effective as, but not superior to, CER in terms of weight loss, BP, body composition, HbA1c, and blood lipid levels in overweight and obese patients with high blood pressure. Our findings showing comparable weight loss in both groups align with a majority of other studies in the field, indicating that IER is neither superior nor inferior to daily calorie restriction. The change in weight loss obtained in our study was comparable to that achieved in a trial conducted by Headland and colleagues in healthy overweight and obese adults (19). Recently, a large meta-analysis including 18 studies has compared the efficacy of intermittent fasting vs. continuous caloric restriction in overweight and obese adults; however, the results remained less conclusive and controversial (22). In our study, both weight loss and BP reduction were greater in a subgroup of obese compared with overweight participants, which means that obese populations benefit more from energy restriction.

Several human studies have been conducted on BP in obese populations, with the majority of studies reporting no differences between the two interventions (15, 23, 24). Indeed, almost all current published research and randomized controlled trials included normotensive participants at baseline, making it difficult to determine between-arm differences in dietary regimens. This study is the first attempt to explore two forms of energy restriction in overweight and obese patients with high BP, and we found that the 5:2 diet is an effective strategy and non-inferior to that of daily calorie restriction for blood pressure and weight control. It is noteworthy that participants appeared to be younger and have a lower BMI in the present trial than in other similar studies. The following reasons may have contributed to this discrepancy: we inferred that the Chinese elderly were less willing than young and middle-aged adults to participate at enrollment due to a lack of health education, and the baseline body weight is much lower in Asians than in Western populations, as investigations have determined (25, 26).

Some evidence suggests that intermittent calorie restriction may be superior to daily calorie restriction for the retention of lean mass at the expense of fat mass (9). In the present study, it also appeared as though a lower proportion of total fat-free mass was lost in response to IER when compared with CER. These findings were consistent with earlier studies, although the reason for the discrepancy in total fat-free mass is not clear. Guerrero et al. have shown greater loss of fat mass in participants who underwent intermittent fasting in three trials with a duration longer than 24 weeks, whereas no significant differences were observed between groups in shorter-duration studies (22). Altogether, these findings suggest that a longer duration of intermittent diet may be beneficial for the retention of lean mass compared with CER.

A randomized non-inferiority trial by Carter et al., determined that IER is an effective alternative dietary regimen for reduction of HbA1c comparable to CER in patients with type 2 diabetes at 12 months, while HbA1c increased to above baseline levels at 24-month follow-up in both groups (20). Likewise, we observed a mild HbA1c decrease at 6 months in our study population. No significant difference in blood lipid levels was identified between the two intervention groups. However, Catenacci et al., observed a significant reduction in total cholesterol and low-density lipoprotein (LDL) cholesterol values in the alternate-day fasting group compared with the CER group in obese adults (27). Worthy of note, alternate-day fasting is another form of IER that consists of a “fast day” (0–25% of caloric needs) alternating with a “fed day” (ad libitum food consumption). Besides, the sample population was relatively small, and all food was provided by the investigators in their study. In contrast, Trepanowski et al.'s study indicated that LDL cholesterol levels were significantly elevated among the participants in the alternate-day fasting group compared with those in the CER group at 12 months (28).

The main strength of this pragmatic trial was that we had made great efforts to replicate the real-world environment. As a result, no meal replacement or foods were supplied. Taking this fact into consideration, the acceptability of the diet protocol was very high, and only nine participants could not comply with the protocol. Both dietary regimens were safe for individuals with type 2 diabetes or high BP or both, as well as for people using insulin and/or sulfonylureas. Both types of calorie restriction require medication changes and periodic monitoring, especially at the beginning stage. No participants in either group experienced a major hypoglycemic episode in our trial. Only 13 participants experienced mild symptomatic hypoglycemia and then contacted their medical practitioner for further medication adjustment.

Our study has some limitations. Although a questionnaire was provided to participants to record the variety and exact amount of their usual food intake, the present study consisted primarily of nutrition education and diet guidelines that did not provide actual foods, which may have impacted the results. Furthermore, the considerable dropout rate limits the generalizability of the results to obese and hypertensive population groups, although we have demonstrated from completers and ITT analyses that the main findings remained significant despite attrition. In addition, we excluded those with extremely high BP and diabetic patients with a previous history of severe hypoglycemia, for which immediate medical attention and safety issues should be taken into account; therefore, diet intervention may not be applicable to individuals with BP > 180/120 mmHg or poorly controlled diabetes. Finally, we did not include a DASH diet control group as we have previously had difficulty in recruiting participants when they were aware that the DASH diet might not be as effective as calorie restriction for weight loss (29).

## Conclusions

In conclusion, a 2-day severe energy restriction with 5 days of habitual eating compared to 7 days of CER provides an acceptable alternative for BP control and weight loss in overweight and obese individuals with hypertension after 6 months. IER may offer a useful alternative strategy for this population, who find continuous weight-loss diets too difficult to maintain. Further studies are needed to determine whether a 5:2 diet is sustainable and effective in the long term compared with CER.

## Data Availability Statement

The raw data supporting the conclusions of this article will be made available by the authors, without undue reservation.

## Ethics Statement

The studies involving human participants were reviewed and approved by the Ethics Committee of Jiaxing University. The patients/participants provided their written informed consent to participate in this study.

## Author Contributions

C-JH: conceptualization, methodology, software, investigation, and writing—original draft. Y-PF: data curation, formal analysis, and writing—original draft preparation. C-YZ: resources, visualization, and investigation. MY: software. GQ: formal analysis, validation, and resources. H-LH: conceptualization, methodology, writing—reviewing and editing, and supervision. C-LZ: project administration, writing—reviewing, and editing. All authors contributed to the article and approved the submitted version.

## Funding

This research was funded by Zhejiang Provincial Basic Public Welfare Research Program of China under Grant No. LGF19H020007, Provincial-Municipal Joint Construction of Key Medical Disciplines In Zhejiang Province (2019-ss-xxgbx), Jiaxing Key Innovation Team Fund (2018-xjqxcxtd), Jiaxing Science and Technology Program under Grant Nos. 2021AD30148, 2021AD30132, and 2020AY30006, Zhejiang Provincial Health Science and Technology Program under Grant No. 2021KY1105, Pioneer Innovation Team of Jiaxing Arteriosclerotic Diseases Research Institute (XFCX-DMYH).

## Conflict of Interest

The authors declare that the research was conducted in the absence of any commercial or financial relationships that could be construed as a potential conflict of interest.

## Publisher's Note

All claims expressed in this article are solely those of the authors and do not necessarily represent those of their affiliated organizations, or those of the publisher, the editors and the reviewers. Any product that may be evaluated in this article, or claim that may be made by its manufacturer, is not guaranteed or endorsed by the publisher.
