# Supplementary material for: Effects of Intermittent Compared With Continuous Energy Restriction on Blood Pressure Control in Overweight and Obese Patients With Hypertension
Source: Front Cardiovasc Med. 2021 Oct 18;8:750714. doi: 10.3389/fcvm.2021.750714 (PMC8558476; doi:10.3389/fcvm.2021.750714)
Supplement: Supplementary file 1 [file Table_1.doc]

**Table S1.** Intermittent energy restriction example meal plan

|  | 2100 kJ/day | 2520 kJ/day |
| --- | --- | --- |
| Breakfast | A piece of toast + a cup of low-fat milk |  |
| Lunch | 100g of cooked chicken breast + 100g low carbohydrate vegetables | Add: a piece of toast or an egg |
| Dinner | 2 egg whites + 100g low carbohydrate vegetables |  |
